# Resolving Structures of Paramagnetic Systems in Chemistry and Materials Science by Solid‐State NMR: The Revolving Power of Ultra‐Fast MAS

**DOI:** 10.1002/anie.202408704

**Published:** 2024-11-11

**Authors:** Jonas Koppe, Kevin J. Sanders, Thomas C. Robinson, Arthur L. Lejeune, David Proriol, Sebastian Wegner, Armin Purea, Frank Engelke, Raphaële J. Clément, Clare P. Grey, Andrew J. Pell, Guido Pintacuda

**Affiliations:** ^1^ Centre de RMN Très Hauts Champs de Lyon (UMR5082—CNRS ENS Lyon UCB Lyon 1) Université de Lyon 5 rue de la Doua 69100 Villeurbanne France; ^2^ IFP Energies Nouvelles Rond-point de l'échangeur de Solaize 69360 Solaize France; ^3^ Bruker Biospin Rudolf-Plank-Str. 23 76275 Ettlingen Germany; ^4^ Yusuf Hamied Department of Chemistry University of Cambridge Lensfield Road Cambridge CB2 1EW United Kingdom; ^5^ Materials Department and Materials Research Laboratory University of California Santa Barbara CA 93106 USA

**Keywords:** solid-state NMR, paramagnetic materials, ultrafast MAS, high-resolution, electronic structures

## Abstract

Ultra‐fast magic‐angle spinning (100+kHz) has revolutionized solid‐state NMR of biomolecular systems but has so far failed to gain ground for the analysis of paramagnetic organic and inorganic powders, despite the potential rewards from substantially improved spectral resolution. The principal blockages are that the smaller fast‐spinning rotors present significant barriers for sample preparation, particularly for air/moisture‐sensitive systems, and are associated with low sensitivity from the reduced sample volumes. Here, we demonstrate that the sensitivity penalty is less severe than expected for highly paramagnetic solids and is more than offset by the associated improved resolution. While previous approaches employing slower MAS are often unsuccessful in providing sufficient resolution, we show that ultra‐fast 100+kHz MAS allows site‐specific assignments of all resonances from complex paramagnetic solids. Combined with more reliable rotor materials and handling methods, this opens the way to the routine characterization of geometry and electronic structures of functional paramagnetic systems in chemistry, including catalysts and battery materials. We benchmark this approach on a hygroscopic luminescent Tb^3+^ complex, an air‐sensitive homogeneous high‐spin Fe^2+^ catalyst, and a series of mixed Fe^2+^/Mn^2+^/Mg^2+^ olivine‐type cathode materials.

## Graphical abstract

Ultra‐fast magic‐angle spinning (MAS) over 100 kHz has transformed solid‐state NMR for biomolecules but struggled with paramagnetic powders due to rotor size and sensitivity issues. Advances now permit routine analysis of sensitive samples, enhancing spectral resolution and overcoming past limitations. This enables precise characterization of paramagnetic systems′ structures and properties, crucial for catalysts and battery materials, demonstrated on various complex materials.

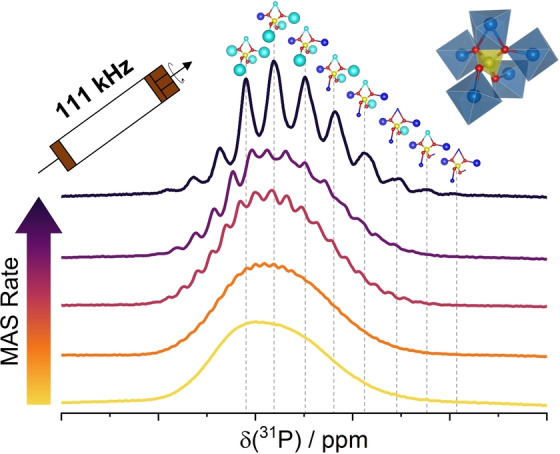

Solid‐state nuclear magnetic resonance (NMR) spectroscopy with magic‐angle spinning (MAS) is a powerful technique for the atomic‐scale characterization of the chemical properties of crystalline, poorly‐crystalline or amorphous solids, with applications throughout the chemical and biological sciences.^[1]^ Many relevant samples contain transition metal ions, and draw their properties from unpaired electrons. For many years, solid‐state NMR studies of such systems were assumed to be intractable, since the magnetic moments of the unpaired electrons perturb the properties of the surrounding nuclei, producing very large shifts and shift anisotropies, together with short relaxation times and large field inhomogeneities,^[2]^ making meaningful NMR spectra unobtainable. However, these same effects contain a direct link to geometry and electronic structure,^[3]^ which provided a strong motivation to overcome the limitations to obtaining and meaningfully interpreting solid‐state NMR spectra of paramagnetic samples.

Progress to date has focused on three main avenues of inquiry: Firstly, the development of theoretical and computational tools necessary for understanding, modeling and interpreting the effects of the unpaired electrons on the nuclei;[^4^, ^5^, ^6^, ^7^, ^8^, ^9^, ^10^] secondly, the design of tailored radio‐frequency (RF) pulse schemes for the excitation and manipulation of the broad signal components;[^11^, ^12^, ^13^] thirdly and most importantly, the engineering of instrumentation capable of faster MAS.[^14^, ^15^, ^16^] MAS is a crucial tool for the majority of solid‐state NMR studies, since it causes the narrowing of the spectral linewidth by averaging the anisotropic spin interactions, and therefore increasing resolution and sensitivity. In paramagnetic systems, the spin interactions, particularly those involving electron‐nuclear couplings, are particularly large, and require MAS rates that are substantially larger than those routinely employed on diamagnetic systems.[^17^, ^18^, ^19^] Substantial progress has been realized by combining MAS frequencies up to 60 kHz with fast adiabatic RF pulses and sophisticated computational methods, opening the way for solid‐state NMR to attack problems in a wide variety of systems, including catalysts,[^18^, ^20^, ^21^] battery materials,[^22^, ^23^, ^24^] metal–organic frameworks,[^25^, ^26^] and metalloenzymes.[^27^, ^28^, ^29^] During the last few years, even faster rates of 100 kHz have become accessible.[^30^, ^31^, ^32^] This has revolutionized the field of biomolecular NMR, since it permits the acquisition of resolved spectra from ^1^H nuclei abundant in biomolecules,[^32^, ^33^, ^34^] but has only rarely found application in materials science.[^35^, ^36^, ^37^, ^38^, ^39^, ^40^, ^41^, ^42^, ^43^, ^44^]

One potential disadvantage of employing faster MAS is that it requires smaller sample holders (rotors), with smaller active sample volumes, resulting in lower sensitivity. However, for uniformly ^13^C,^15^N‐labelled diamagnetic biomolecules, the lost sensitivity can be overcome by direct detection of the more sensitive ^1^H instead of the more conventional ^13^C nucleus.^[34]^ This ^1^H detection scheme has been straightforwardly extended to paramagnetic biomolecules, permitting the localization of the metal center in a metalloprotein with picometer resolution.^[45]^

Obviously, this scheme cannot be implemented for inorganic materials that do not contain H atoms. Moreover, while for small organometallic molecules natural abundance ^1^H spectra can be obtained, ^1^H detection is not always a possible route to obtain the spectrum of e. g., ^13^C, partly due to the low abundance of the heteronuclei and the inefficiency of coherence transfers in paramagnetic systems containing a dense network of metal ions.

In parallel, the dense network of paramagnetic metal ions induces substantial inhomogeneous broadening, which is not reduced by MAS. The low sensitivity and large inhomogeneous broadening have therefore proven to be the main blockage to the widespread uptake of 100 kHz MAS technology for studying these materials.

Moreover, the experience of the solid‐state NMR community is that the smaller rotors are more cumbersome to handle during the steps of sample preparation, and prohibitively so when these steps are to be performed under an inert atmosphere in a glovebox—which is generally difficult to avoid when looking at most materials extracted from batteries. This is exacerbated by the fragility of the rotors, which have thinner walls than those of larger diameters. As a result, the routine application of ultra‐fast MAS to sensitive samples is perceived as impracticable. However, we have overcome these latter problems through the development of a ceramic material production process tailored to the specific requirements of fast‐spinning rotors, such as low defect density in the rotor material and improved dimensional precision of the machined rotors. These new ceramics, along with additional test procedures, have resulted in rotors that are stronger and less prone to fracture (see Section S2 in the Supporting Information for details). These improvements are complemented by adapted tools and procedures for sample packing, and the new generation of rotors is now commercially available, providing enhanced reliability for fast MAS applications. These developments appear to us as one key for the adoption of fast MAS by the chemist, where investigations on a large number of often air/moisture‐sensitive samples are typically required.

This allows us to focus on the first hurdle, i. e., low sensitivity and resolution. Here we show that by applying 100+kHz MAS to paramagnetic organic and inorganic materials we can observe spectral features from some nuclei that would otherwise be undetectable at lower MAS.

The benefits of fast MAS are showcased on the hygroscopic terbium (4f^8^, J=6) tris‐dipicolinate complex Na_3_[Tb(DPA)_3_], the ^1^H solid‐state NMR spectra of which are shown in Figure 1. Figure 1a shows three 1D spectra acquired at three different MAS frequencies using state‐of‐the‐art broadband excitation pulse sequences,^[11]^ namely the double‐adiabatic spin‐echo sequence (more details of which are provided in the Supporting Information). The three chosen spinning frequencies are 30, 60 and 111 kHz, which represent typical maximum operating frequencies for three widely‐used MAS probes (2.5, 1.3, and, 0.7 mm, respectively) within the community. All three spectra were actually acquired with the 0.7 mm probe, in order to highlight the staggering gain in sensitivity achieved by increasing spin rate from 30 to 111 kHz, independently from the decrease in the sample volume. In addition, the three spectra were acquired with increased RF field amplitudes (from 100 kHz at 30 kHz MAS to 435 kHz at 111 kHz MAS), which represents the maximum applicable non‐selective pulse amplitudes for the coils in the different probes (also see Figure S5 for a comparison at identical RF field strengths).

**Figure 1:**
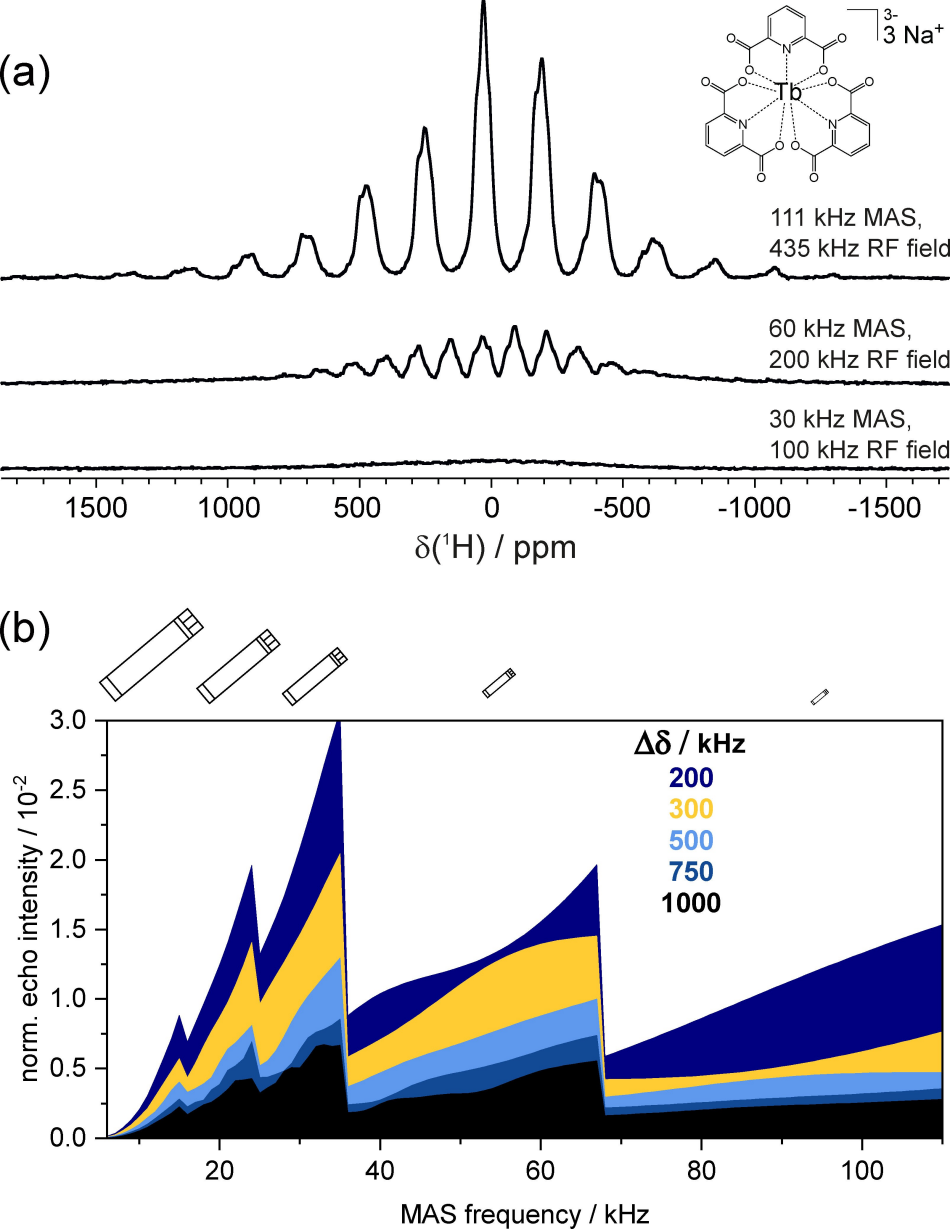
Effect of faster MAS on resolution and sensitivity for a highly paramagnetic organometallic microcrystalline powder. (a) Molecular structure and ^1^H MAS NMR spectra of Na_3_[Tb(DPA)_3_] obtained at 11.75 T, at spinning frequencies from 30 to 111 kHz. The spectra were acquired using a double spin‐echo employing a conventional π/2 excitation pulse and a pair of short high‐power adiabatic pulses, of RF‐field strengths representative of the maximum applicable for a 2.5 mm, 1.3 mm, and 0.7 mm probe. (b) Numerical simulation[^46^, ^47^] of the normalized echo intensities for the isotropic peak of MAS NMR signals with shift anisotropies Δδ increasing from 200 to 1000 kHz. The asymmetry parameter is set to η=1 , so that the width of the spinning side‐band manifold is 2Δδ . The echo intensity is calculated from the volumes and filling factors of different rotor sizes (4 to 0.7 mm outer diameters), MAS averaging of the shift anisotropy, variation of the coherent linewidth and homogeneous signal dephasing during two rotor periods. The latter two are calculated assuming a coherence lifetime $T_{2}^{'}$ that ranges from ~50 μs at 5 kHz MAS to ~500 μs at 111 kHz. The intensities are scaled relative to the absolute signal intensity from a sample in a 4 mm rotor in the absence of a shift anisotropy and $T_{2}^{'}$ dephasing. Further details regarding experimental parameters from (a), and various factors for the normalized echo intensity in (b) are provided in Supporting Information.

We see that 30 kHz MAS is wholly inadequate, since there is no resolution of either the individual ^1^H environments or the spinning side‐bands, and the maximum RF field is at too low power to even excite the full width of the spectrum at this field strength. Whilst we do see an improvement on all these three points on increasing the MAS frequency to 60 kHz,[^48^, ^49^] it is only at 100+kHz that the side‐bands are baseline resolved and that the full pattern spanning approximatively 1.5 MHz is excited. Notably, there is no signal due to water as the sample was packed in a glovebox and the 0.7 mm rotor is sufficiently air‐tight to prevent subsequent uptake of moisture. The sensitivity gain from increased MAS is due to a combination of three factors: concentrating the signals into fewer side‐bands, minimizing relaxation and coherent homogeneous dephasing losses during progressively shorter spin‐echo delays (see Figure S4), and more efficient broadband excitation. However, in practice, the spectra acquired at 30 and 60 kHz would actually be acquired using larger outer diameter rotors (2.5 and 1.3 mm, respectively), which have smaller filling factors but larger active volumes than the 0.7 mm rotors, and therefore are associated with intrinsically higher sensitivity. The actual sensitivity is therefore a trade‐off between the increase from faster MAS and a large reduction from the decrease in the rotor size. This is represented in Figure 1b, which incorporates all of these factors into determining the sensitivity versus MAS frequency for coherence lifetimes ($T_{2}^{'}s$ ) and shift anisotropies commonly encountered for paramagnetic molecules. For shift anisotropies ranging from 200 to 1000 kHz, the loss in sensitivity for a 0.7 mm rotor at 111 kHz MAS versus a 1.3 mm rotor at 67 kHz MAS is between a factor of 1 and 2, despite the reduction of the volume by a factor of ~4 (and between a factor of 2 and 3, despite the reduction of the volume by a factor of ~23, when comparing to a 2.5 mm rotor at 35 kHz MAS). This relatively modest penalty is more than offset by the huge potential gain in resolution. This is illustrated in Figure 2, which shows the ^1^H NMR spectra of a powdered sample of the air‐sensitive homogeneous catalyst N‐(diisopropylphosphino)‐N‐methylpyridin‐2‐amine‐dichloro‐iron(II) (3d^6^, S=2) complex (Fe(py‐NMe‐PiPr_2_)Cl_2_).^[50]^ This molecule is a more exacting test for MAS NMR, featuring eleven different ^1^H environments in the crystal lattice. Again, we see a steady improvement in resolution at higher MAS, with more signals becoming progressively resolved on increasing from 20 to 111 kHz. At 111 kHz, eight distinct signals can be clearly identified, including three very weak signals at −37, 109 and 283 ppm, which are invisible at 60 kHz MAS and below.^[50]^ The increase in spectral quality can be exploited by performing more sophisticated experiments in order to fully separate the individual signals and extract their site‐specific shift tensor parameters and completely assign them to proton sites in the molecule. Specifically, we acquired a 2D adiabatic magic‐angle turning (aMAT)^[22]^ spectrum which separates signals by suppressing the side‐band manifold structure in an “infinite MAS” indirect dimension. The resulting “infinite MAS” spectrum and traces of the spinning side‐band manifolds are shown in Figure 2b. Eight individual signals are resolved, corresponding to all the distinct ^1^H sites, with the exception of the four isochronous methyls of the two iso‐propyl groups. Interestingly, the three signals (at −37, 109 and 283 ppm) with the largest shift anisotropies (580–780 ppm) escape detection at 60 kHz MAS and so were not reported in a previous study^[50]^ (see Section S7). These results demonstrate a new atomic‐level insight into organometallic complexes at natural abundance which is expected to boost structure‐based investigations of the many facets of transition metal chemistry.

**Figure 2:**
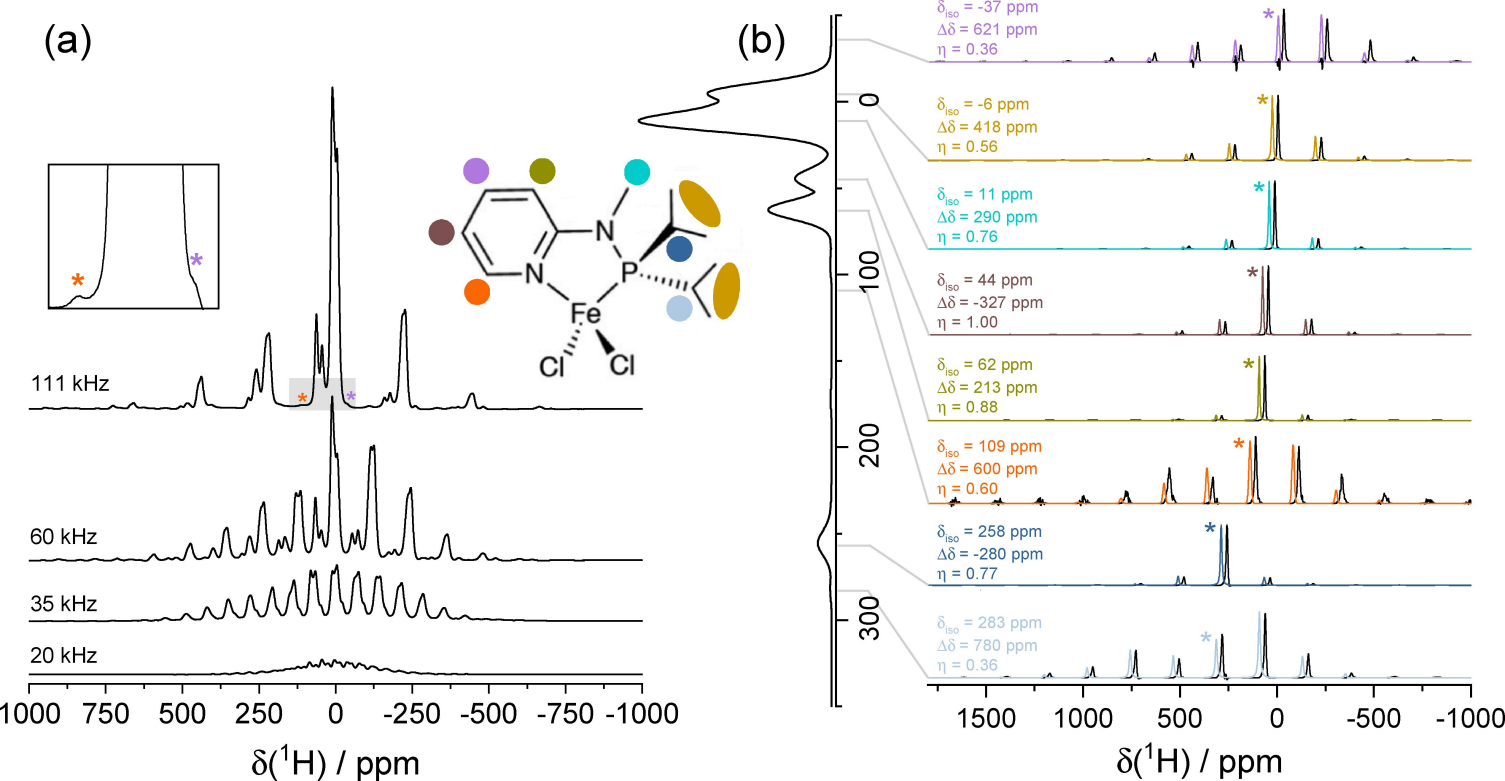
Increase in resolution and consequent characterization of the ^1^H NMR spectra of a microcrystalline organometallic catalyst at faster MAS. (a) Structure and 1D ^1^H MAS NMR spectra of Fe(py‐NMe‐PiPr_2_)Cl_2_ obtained at 11.75 T, using spinning frequencies increasing from 20 to 111 kHz. Asterisks denote signals that are only visible at 111 kHz MAS. (b) Signal traces extracted from the 2D aMAT at 111 kHz MAS and fitted to obtain the shift tensor parameters.^[46]^ Asterisks denote the isotropic peaks and the color code refers to the molecular structure in (a). Simulated signals are offset horizontally by +30 ppm for clarity. Also shown vertically in (b) is the “infinite‐MAS” spectrum obtained from the projection of the 2D aMAT. Further experimental details are summarized in the Supporting Information.

The benefits of faster MAS are also evident for additional research fields in the chemical sciences, as demonstrated here on inorganic materials. Specifically, we present an application to a series of lithium‐ion cathode materials based on the classic olivine‐type LiFePO_4_, originally proposed by Goodenough et al.^[51]^ (Figure 3a). Potential improvement in battery performance can be realised by replacing the Fe with a solid solution of other metal ions, e. g., Fe/Mn or Mn/Mg. There has been a tremendous resurgence of work on doped olivines, as LiFePO_4_ is now used commercially in an increasingly wide range of applications including in micro‐grids and in electric vehicles.[^52^, ^53^, ^54^, ^55^, ^56^, ^57^] Understanding the full battery structure and how it changes during electrochemical cycling requires us to monitor changes at the atomic scale. In principle this can be done via ^31^P solid‐state NMR, but requires us to resolve all the individual local environments, which is extremely challenging for these solid‐solution materials.

**Figure 3:**
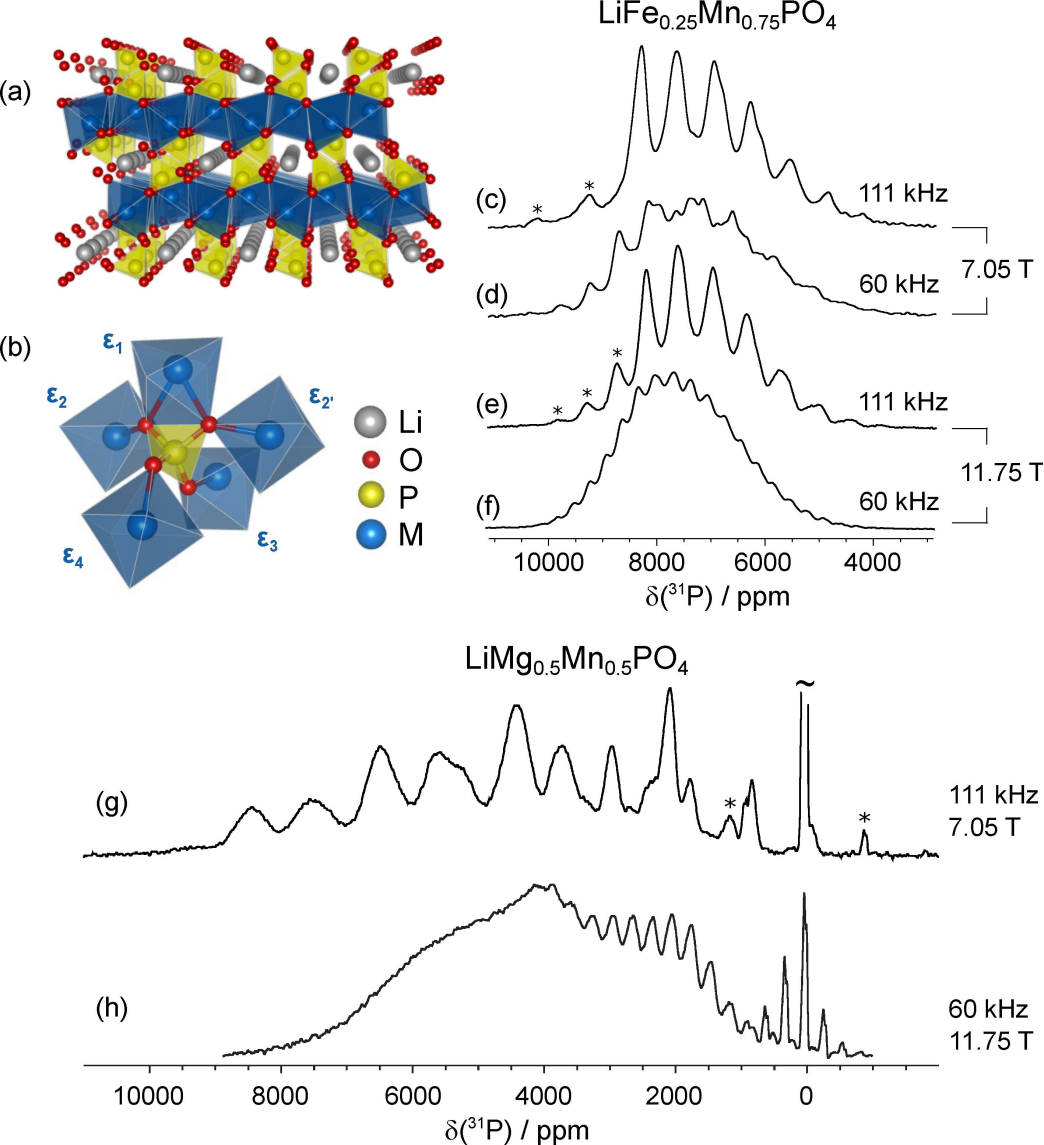
Effect of faster MAS on the resolution for a highly paramagnetic inorganic microcrystalline battery material. (a) 3D lattice structure of olivine‐type LiMPO_4_ (M is a divalent metal ion). (b) Local coordination environment of the P sites. The total ^31^P shift is defined by the metal species occupying each of the five nearest neighbor metal sites and is given by the sum of five pathway contributions ϵ_1,2,2’,3,4_. (c–f) ^31^P MAS NMR spectra of LiFe_0.25_Mn_0.75_PO_4_ obtained at all four possible combinations of the pair of magnetic fields 7.05 T and 11.75 T, and MAS frequencies of 60 and 111 kHz. (g–h) ^31^P MAS NMR spectra of LiMg_0.5_Mn_0.5_PO_4_ obtained at 7.05 T and 111 kHz MAS (g), and at 11.75 T and 60 kHz MAS (h). Unambiguously identifiable spinning sidebands are marked with asterisks in all spectra. Further experimental details are summarized in the Supporting Information.

Initially, we focus on LiFe_0.25_Mn_0.75_PO_4_ where there are thirty‐two distinct local ^31^P environments defined by the occupation of the five nearest‐neighbour transition metal sites by either Fe^2+^ (high spin 3d^6^) or Mn^2+^ (3d^5^) (Figure 3b). This material was previously characterised by a combination of solid‐state NMR at 11.74 T and 60 kHz MAS and density‐functional theory (DFT) computations. A full assignment was impossible from 1D data alone and required a series of 2D aMAT spectra from samples of varying compositions of Fe/Mn.^[22]^ A 1D spectrum acquired under the same conditions (11.74 T and 60 kHz MAS) is shown in Figure 3f, and clearly demonstrates the shortcomings of slower MAS. The previous characterisation concluded that, of the contributions to the ^31^P shifts from the five neighbouring transition metal ions, two are equivalent (ϵ_2_ and ϵ_2_’ in Figure 3b), resulting in a total of twenty‐four distinct resonances. These resonances are not resolved at 11.74 T and 60 kHz MAS due to the broadening from the shift anisotropies presenting as overlapping spinning‐sideband manifolds and inhomogeneous side‐band broadening. Under these experimental conditions, partial resolution of these resonances is achievable only with the application of aMAT. The result is that the twenty‐four resonances are separated into eight distinct groups, the range of isotropic shifts being within the linewidths for each group.

A classical solution to this problem, to employ a lower field to reduce the size of the anisotropic shifts, is here insufficient as shown by spectrum acquired at 7.05 T and 60 kHz MAS (Figure 3d). For both fields, faster MAS at 111 kHz enables a clear separation of the groups of resonances (the only absent resonance being from the low‐populated all‐Fe environment at ~3800 ppm) (Figure 3c and 3e). The side‐band structure is suppressed to such an extent that the spectra are dominated by the isotropic resonances (as demonstrated by a comparison with a 2D aMAT spectrum provided in the Supporting Information in Figure S9). The 1D spectrum is now of sufficient quality that it can be assigned directly. This was done by comparison with the DFT‐calculated shifts for the twenty‐four distinct local ^31^P environments and a simple fitting model that interprets each shift as the sum of contributions from the five nearest neighbor metal ions (full details are given in Section S9 of the Supporting Information). The fully‐assigned spectrum is shown in Figure 4a. Since the isotropic shifts are almost entirely due to the Fermi‐contact interaction, size and sign of each individual shift are proportional to the electron density transferred from the five metal ions to the P *s*‐orbitals. The assignment allows to quantify directly the difference in the transferred electron density for Mn vs Fe for each metal site. The spectra integrals reported by the fit are also consistent with Mn/Fe being randomly distributed throughout the lattice in a solid solution and rule out the presence of any compositional ordering of these metal ions.

**Figure 4:**
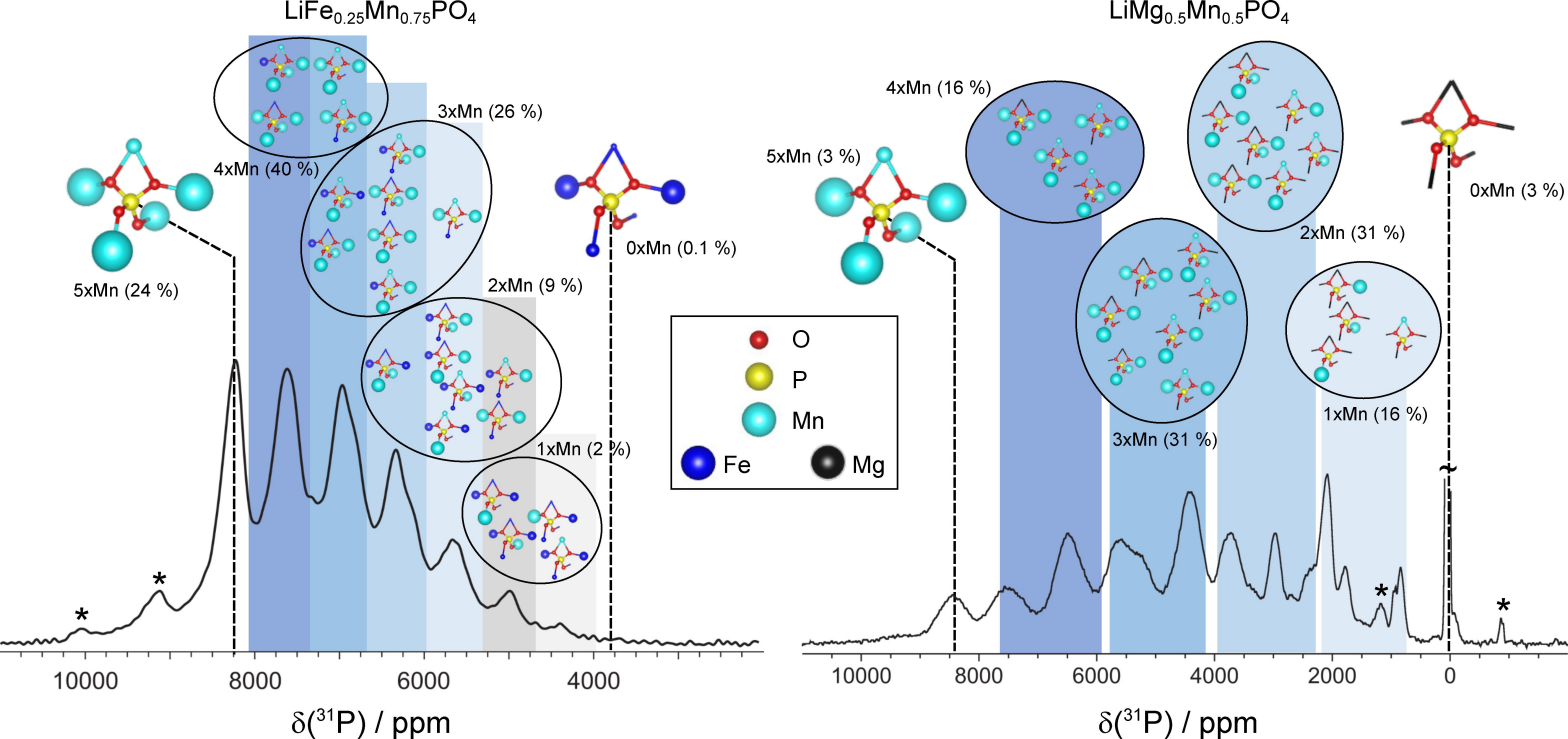
Complete characterization of the local P environments of mixed‐composition olivine‐type LiMPO_4_ battery materials. (a–b) ^31^P MAS NMR spectra of LiFe_0.25_Mn_0.75_PO_4_ and LiMg_0.5_Mn_0.5_PO_4_, respectively, obtained at 7.05 T and 111 kHz MAS and the assignment of the peaks as groups of resonances from the twenty‐four distinct environments. In each case, the horizontal position of the P atom marks the assigned ^31^P shift of the corresponding local environment. In the depiction of the local environments, the relative sizes of the spheres representing the metal ions reflect the corresponding absolute pathway contributions, i. e., the isotropic ^31^P shift that is induced by replacing the diamagnetic Mg^2+^ with either Fe^2+^ or Mn^2+^ at the respective position. The fractional populations of each group of local environments, assuming a random distribution of the two metal ions in the lattice, are given in percent and match the signal integrals indicating that the composition is a solid solution. Further experimental details are summarized in Supporting Information.

MAS at 111 kHz comes into its own when we turn our attention to the mixed phases LiMg_*x*_Mn_1‐*x*_PO_4_. Spectroscopically, these materials are the most demanding presented here, as shown in Figure 3g and 3h. Figure 3h presents the ^31^P spectrum of LiMg_0.5_Mn_0.5_PO_4_ at 60 kHz MAS and 11.74 T, which exhibits a spectral dispersion of more than 8000 ppm, twice that for the mixed Fe/Mn phases. The two main problems with spectral acquisition and interpretation are that there are again twenty‐four overlapping resonances with broad anisotropic patterns and therefore no resolution, even with the larger dispersion, and that excitation of the full spectrum is right at the limit of what is possible with state‐of‐the‐art broadband NMR techniques and practical RF fields in 1.3 mm coils. The resulting spectrum is a mostly featureless broad line, impossible to interpret. The combination of a lower magnetic field and faster MAS solves both problems.

The spectrum in Figure 3g demonstrates the distinct improvement in spectral quality on combining 111 kHz MAS with the higher RF strengths available from 0.7 mm coils and a lower magnetic field (7.05 T). For this sample, due to larger ^31^P frequency dispersion, the signals due to the different local environments group into fourteen distinct spectral regions, with reduced overlap. This 1D spectrum can now be completely assigned, as shown in Figure 4b. The assigned shifts allow the *absolute* unpaired electron density from each of the Mn to the P in each of the distinct environments to be extracted, in contrast to the interpretation of the spectra of the mixed Fe/Mn phases, where only values *relative* to Fe can be extracted. In addition, the spectral integrals once again are consistent with a random distribution of Mn/Mg throughout the lattice.

These additional results show that solid‐state NMR with faster MAS is an ideal tool for studying the finer structural details of paramagnetic inorganic materials, in particular for obtaining information on details that are not readily available using other structure characterization techniques, such as the ordering of atomic species within mixed composition materials.

In this communication we have outlined how the main blockages to applying 100 kHz MAS technology to highly paramagnetic organometallic and inorganic materials have been overcome. In particular, the technology for constructing and using the 0.7 mm rotors is now mature, and approaches have been developed to allow more straightforward packing of the tiny rotors, allowing a more routine analysis of air/moisture‐sensitive samples. The loss in sensitivity associated with the smaller sample volumes is less than would be superficially expected and is more than compensated by the substantial improvement in resolution, due to the more complete suppression of the spinning‐sideband manifold, even in the presence of large shift anisotropies and inhomogeneous broadenings typical of these paramagnetic systems. The examples given here additionally demonstrate that the assignments and interpretations of NMR data for many highly paramagnetic systems are only possible when we employ faster MAS. The rapid acquisition of interpretable NMR spectra of paramagnetic systems, without the need of more complex and time‐demanding 2D pulse schemes, enabled by fast MAS as described here, transforms solid‐state NMR into a routine method for structure investigation in chemistry and materials science.

## Supporting Information

The Supporting Information contains details on the synthesis of the samples used, on the technological progress for the 0.7 mm MAS rotors, and all experimental NMR parameters. A detailed analysis of the sensitivity of 100 kHz MAS, spectral filters for processing 2D aMAT spectra, and modelling routines is also provided.

## Conflict of Interests

The authors declare no conflict of interest.

## Supporting information

As a service to our authors and readers, this journal provides supporting information supplied by the authors. Such materials are peer reviewed and may be re‐organized for online delivery, but are not copy‐edited or typeset. Technical support issues arising from supporting information (other than missing files) should be addressed to the authors.

Supporting Information

## Data Availability

The data that support the findings of this study are openly available in Zenodo at https://doi.org/10.5281/zenodo.11120901, reference number 11120901.
