# Supplementary material for: Resolving Structures of Paramagnetic Systems in Chemistry and Materials Science by Solid‐State NMR: The Revolving Power of Ultra‐Fast MAS
Source: Angew Chem Int Ed Engl. 2024 Nov 11;64(1):e202408704. doi: 10.1002/anie.202408704 (PMC11701365; doi:10.1002/anie.202408704)
Supplement: Supplementary file 1 — Supporting Information [file ANIE-64-e202408704-s001.pdf]

Angewandte Chemie
Eine Zeitschrift der Gesellschaft Deutscher Chemiker

Supporting Information

Resolving Structures of Paramagnetic Systems in Chemistry and Materials Science by Solid-State NMR: The Revolving Power of Ultra-Fast MAS

J. Koppe, K. J. Sanders, T. C. Robinson, A. L. Lejeune, D. Proriol, S. Wegner, A. Purea, F. Engelke, R. J. Clément, C. P. Grey, A. J. Pell, G. Pintacuda**

Supporting Information

Resolving structures of paramagnetic systems in chemistry and materials science by solid-state NMR: the revolving power of ultra-fast MAS

Jonas Koppe,[1,#] Kevin J. Sanders,[1,#] Thomas C. Robinson,[1] Arthur L. Lejeune,[1,2] David Proriol,[2] Sebastian Wegner,[3] Armin Purea,[3] Frank Engelke,[3] Raphaële J. Clément,[4,5] Clare P. Grey,[4] Andrew J. Pell,[1,*] Guido Pintacuda[1,*]

[1]Centre de RMN Très Hauts Champs de Lyon (UMR5082 - CNRS, ENS Lyon, UCB Lyon 1), Université de Lyon, 5 rue de la Doua, 69100 Villeurbanne, France

[2]IFP Energies Nouvelles, Rond-point de l'échangeur de Solaize, 69360 Solaize, France

[3]Buker Biospin, Rudolf-Plank-Str. 23, 76275 Ettlingen, Germany

[4]Yusuf Hamied Department of Chemistry, University of Cambridge, Lensfield Road, Cambridge, CB2 1EW, United Kingdom

[5]Materials Department and Materials Research Laboratory, University of California, Santa Barbara, CA 93106, USA

# Contributed equally to this work

* Corresponding authors: andrew.pell@ens-lyon.fr and guido.pintacuda@ens-lyon.fr

Table of Contents

	Description	Page
S1.	**Materials**	S3
S2.	**0.7 mm MAS rotor ceramics, supporting tools, frictional heating, and additional hardware requirements**	S5
S3.	**Experimental NMR Parameters**	S8
S4.	**Sensitivity of 100+ kHz MAS for Paramagnetic Solids**	S11
S5.	**^{1}H MAS NMR Spectra of $Na_3[Tb(DPA)_3]$ at Various RF Fields**	S12
S6.	**2D aMAT NMR Spectra of $Fe(py\text{-}NMe\text{-}PiPr_2)Cl_2$ at 60 and 111 kHz MAS**	S13
S7.	**Spectral Filter for 2D aMAT NMR Spectra at 100+ kHz MAS**	S14
S8.	**Residual Sideband Intensity for $LiFe_{0.25}Mn_{0.75}PO_4$ at 111 kHz MAS**	S16
S9.	**Routine for Modelling the ^{31}P NMR Spectra of $LiMg_xMn_{1-x}PO_4$**	S17
S10.	**References**	S20

S1. Materials

N-(diisopropylphosphino)-N-methylpyridin-2-amine-dichloro-iron(II)

General Considerations

Unless stated otherwise, all reactions were carried out under an atmosphere of argon using standard Schlenk techniques. Solvents were purified and degassed by using standard procedures. All reagents were purchased from commercial suppliers and used without further purification. FT-IR spectra were recorded in the solid state by an ATR Golden Gate (Specac) on a PerkinElmer spectrum one spectrometer. Elemental analyses were determined at London Metropolitan University. NMR spectra were recorded using a Bruker Avance 300 MHz spectrometer equipped with Avance III HD console and a 5 mm BBFO Z-gradient probe.
Chemical shift ^{1}H scales were reported in parts per million and calibrated against the CD_2Cl_2 signals (5.32 ppm for residual ^{1}H). Information for each resonance is reported in the following order: chemical shift (multiplicity, proton assignments and integration) Spectral multiplicity abbreviations are as follows: d, doublet; m, multiplet. Integration refers to the number of protons per signal. Chemical shift ^{13}C scales were reported in parts per million and calibrated against the CD_2Cl_2 signals (53.84 ppm for residual ^{13}C). Chemical shift ^{31}P scales were reported in parts per million and recorded against an external standard of 85 % H_3PO_4 in 10 % D_2O.

Synthesis of $FeCl_2(THF)_{1.5}$

Anhydrous $FeCl_2$ (5 g) was placed into a Soxhlet paper cartridge. A round bottom flask was charged with THF (150 mL) prior to be connected to the Soxhlet apparatus under argon. After 3 days of reflux a suspension of a white solid in a brown solution was obtained. The solid was filtered and washed with THF (2x30 mL) and dried under reduced pressure giving 6.77 g (73 %) of a white powder. Anal. found (calcd.) for $FeCl_2 \cdot 1.5C_4H_8O$: C, 30.6 (30.7); H, 4.99 (5.15). IR (solid): ν(C-O-C THF): 875, 1023 cm^{-1}.

Synthesis of the N-(diisopropylphosphino)-N-methylpyridin-2-amine (py-NMe-P*i*Pr_2)

The synthesis of the ligand was performed following the procedure described by Gambarotta's group.[1] 2-methylaminopyridine (1.0 mL, 9.80 mmol, 1.00 eq.) was dissolved in diethylether (30 mL) and the solution was cooled to -78 °C. n-BuLi in 2.5N in hexanes (6.30 mL, 10.1 mmol, 1.03 eq.) was added dropwise to yield a light yellow solution which was then stirred for 1 h at -78 °C and for 2 h at ambient temperature. Diisopropylchlorophosphine (1.6 mL, 10.1 mmol, 1.03 eq.) was then added dropwise at 0 °C, and the reaction mixture was stirred overnight. The white suspension was transferred on an alumina column and washed with diethylether. The solvents were evaporated under reduced pressure to dryness offering 1.90 g (86 %) of a colorless oil.
^{1}H NMR (300 MHz, CD_2Cl_2, 300 K) δ(ppm): 8.15 (m, $CH_{py\ ortho}$, 1H), 7.5 (m, $CH_{py\ meta}$, 2H), 6.64 (m, $CH_{py\ para}$, 1H), 3.08 (d, CH_3 Me, 3H), 2.2 (m, CH_{iPr}, 2H), 1.15 (m, $CH_{3\ iPr}$, 6H), 1.02 (m, $CH_{3\ iPr}$, 6H). $^{13}C\{^{1}H\}$ NMR (75 MHz, CD_2Cl_2, 300 K) δ(ppm): 163.0, 147.6, 136.4, 113.8, 111.4, 33.1 (br. s.), 26.7, 19.8, 19.5.
$^{31}P\{^{1}H\}$ NMR (121.5 MHz, CD_2Cl_2, 300 K) δ(ppm): 71.7 (br.s).

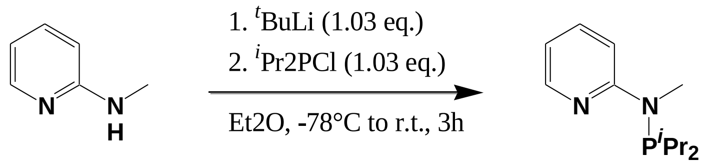

Scheme 1. Synthesis of the py-NMe-PiPr$_2$ ligand.

Synthesis of the FeCl$_2$[py-NMe-PiPr2)]

The py-NMe-P*i*Pr$_2$ ligand (0.674 g, 3.01 mmol, 1.05 eq.) and FeCl$_2$(THF)$_{1.5}$ (0.674 g, 2.87 mmol, 1.00 eq.) were suspended in toluene (20 mL) and stirred for two days at room temperature. A white solid precipitate was formed, which was subsequently filtered, washed twice with diethylether (30 mL) and dried under reduced pressure giving 0.917 g (91 %) of a white solid. Anal. found (calcd.) for $C_{13}H_{22}Cl_2FeNP$: C, 41.05 (41.1), H, 6.12 (6.03), N, 7.82 (7.98).

Sodium terbium tris-dipicolinate

In a 50 mL round bottom flask, dipicolinic acid (3 eq) and Na_2CO_3 (3 eq) were suspended into water (15 mL) and the reaction was stirred until everything dissolved, then $LnCl_3 \cdot 6H_2O$ (1 eq) was added and the reaction was stirred at room temperature for two more hours. The water was evaporated and the white solid was dissolved in the minimum of boiling water. The complex was purified by three successive crystallizations at 4°C conducted in D_2O, resulting in the formation of crystals of general formula $Na_3[Tb(DPA)_3] \cdot 16D_2O$.

Mixed-metal lithium phosphates

$LiFe_{0.25}Mn_{0.75}PO_4$, $LiMg_{0.5}Mn_{0.5}PO_4$ and $LiMg_{0.2}Mn_{0.8}PO_4$ were prepared by hydrothermal synthesis by using $FeSO_4 \cdot 7H_2O$, or $MgSO_4 \cdot 6H_2O$, $MnSO_4 \cdot H_2O$ (both 98% Fisher), H_3PO_4 (85 wt % solution Fisher) and LiOH (98% Aldrich) in the stoichiometric ratios 0.25:0.75:3.0:1.0, 0.5:0.5:3.0:1.0, and 0.2:0.8:3.0:1.0, as described previously in the literature.[2] $MnSO_4 \cdot H_2O/FeSO_4 \cdot H_2O$ and H_3PO_4 were mixed first, and then 1.3 g/L of sugar and/or L-ascorbic acid (99% Aldrich) was added as an *in situ* reducing agent to minimize the oxidation of Fe^{2+}. Multiwall carbon nanotubes (95% Aldrich), 0.8 g/L, were also added. After LiOH addition, the resulting gel was heated at 180–200 °C for 5 h to 2 days, and the precipitates were collected by suction filtration and dried at 60 °C for 3 h under vacuum.

S2. 0.7 mm MAS rotor ceramics, supporting tools, frictional heating, and additional hardware requirements

The development of new zirconia ceramics for MAS rotors involved improving several key steps in the manufacturing process:

1. **Green Rotor Blanks:** The production of the green rotor blanks was optimized by refining the high-pressure profile, which significantly reduced the presence of voids in the ceramic material. These voids can act as weak points that lead to fractures after the sintering process, so their reduction is critical for improving rotor durability.
2. **Sintering Process:** The sintering process itself was revised to achieve lower failure rates. This included testing different temperature profiles, which yielded better results in terms of reducing defects and improving material strength.
3. **Mechanical Stress Testing:** The robustness of the MAS rotor ceramics was assessed through mechanical stress tests, focusing on their flexural strength. For example, a 0.7 mm MAS rotor spinning at 111 kHz experiences a centrifugal pressure of about 265 MPa on its walls, with a resulting hoop stress of around 330 MPa. These stress values were compared with failure rates predicted by the Weibull probability distribution, which characterizes the likelihood of material failure under stress (sigmoid functions in **Figure S1**). The rotor operation point at high MAS speeds must be positioned in the left range of the Weibull curve with low-failure probability. The stress value of 330 MPa corresponds to an extremely low failure rate of 0.002% using the improved manufacturing process, ensuring a sufficient safety margin for safe operation at high MAS speeds.
4. **Improved Longevity:** Rotors produced using this updated process demonstrated significantly longer lifetimes in actual NMR experiments compared to those made with previous methods. This improvement in manufacturing quality is a key factor in enhancing the reliability of 0.7 mm MAS rotors, alongside careful handling during sample preparation. This refined process not only enhances rotor strength but also provides users with more reliable performance, especially at high spinning speeds.

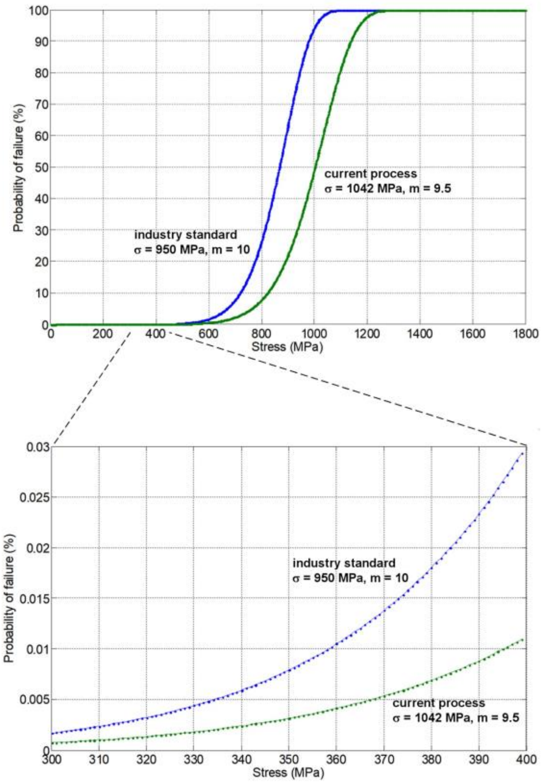

Figure S1. Weibull distribution curves for yttrium-stabilized zirconia ceramics, characterized by the Weibull parameters σ (flexural strength) and *m* (Weibull module). Right-shift of the curves corresponds to larger stability of the ceramic material. The “industry standard” curve represents an average value to be expected from ceramics manufacturers. The “current process” (σ = 1042 MPa, *m* = 9.5) results in improved stress resistance (flexural strength) compared to the industry standard σ = 950 MPa, *m* = 10 curve and is currently used by Bruker to manufacture MAS rotors. The Weibull curve parameters were determined experimentally on rotor blanks with a mechanical stress test.

Notwithstanding the importance of reliable and robust rotor ceramics, the handling of rotors and caps and MAS samples before and after the experiments is of equal relevance for the durability of MAS rotors. A variety of tools to manipulate 0.7mm MAS rotors and caps as well as tools for filling samples has been developed and are commercially available. In particular, these tools facilitate handling air/moisture-sensitive samples. For this purpose, only a small collection of the tools together with the pre-prepared rotor and caps must be introduced into the glovebox. This minimum-required setup to pack an appropriately pre-prepared rotor in a glovebox is shown in **Figure S2**a, comprising the top-cap holder loaded with a top cap (with its wings facing downwards), the sample packer, and the assembly of bottom-cap holder, rotor clamp, main-guidance tool, filling funnel, and the empty rotor with assembled bottom cap. After the rotor is carefully filled leaving sufficient space for the top cap, the filling funnel can be removed, and the assembly turned upside down onto the top-cap holder to close the rotor easily.

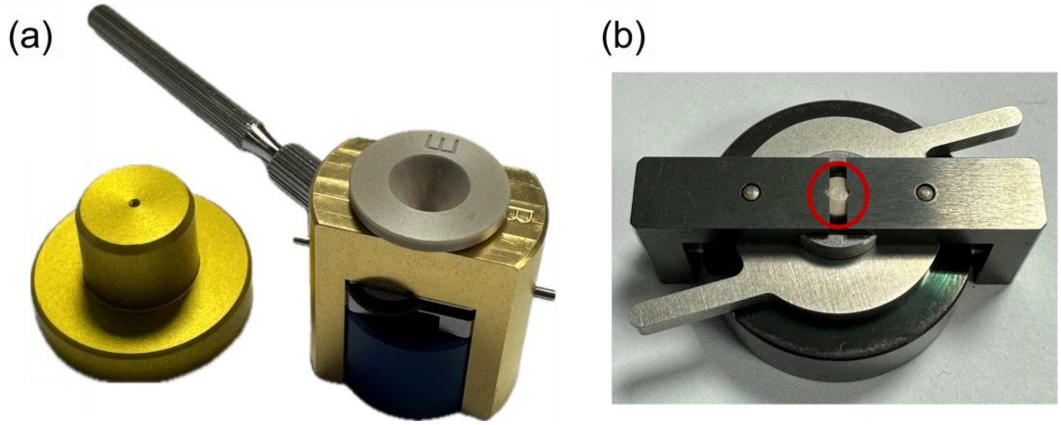

Figure S2. (a) Minimum-required setup to pack and close a 0.7 mm rotor in the glovebox. (b) Rotor-cap removal tools in open state, showing the spring-loaded Teflon plate (red circle), allowing the gentle removal of the caps.

One major improvement on these tools was the introduction of a new version of the rotor-cap removal tool. This now incorporates a spring-loaded Teflon plate (marked with a red circle in **Figure S2**b), which enables to gently induce pressure on the rotor caps to place the rotor in the correct position to remove (in particular top) caps without any damage. With this development, top and bottom caps can now in principle be used multiples times. We however note, that this crucially depends on the operator caution in handling these caps, and rotor caps must always be carefully investigated using the microscope, which is a simple, but in our opinion mandatory tool for handling smaller rotors and caps.

We note that spinning close to, or at the recommend MAS-rate limit for the respective rotor sizes induces considerable frictional heating, as demonstrated in **Figure S3**a. Since paramagnetic shifts are temperature dependent, **Figure S3**b suggests a strategy to cope with potential frictional-heating effects.

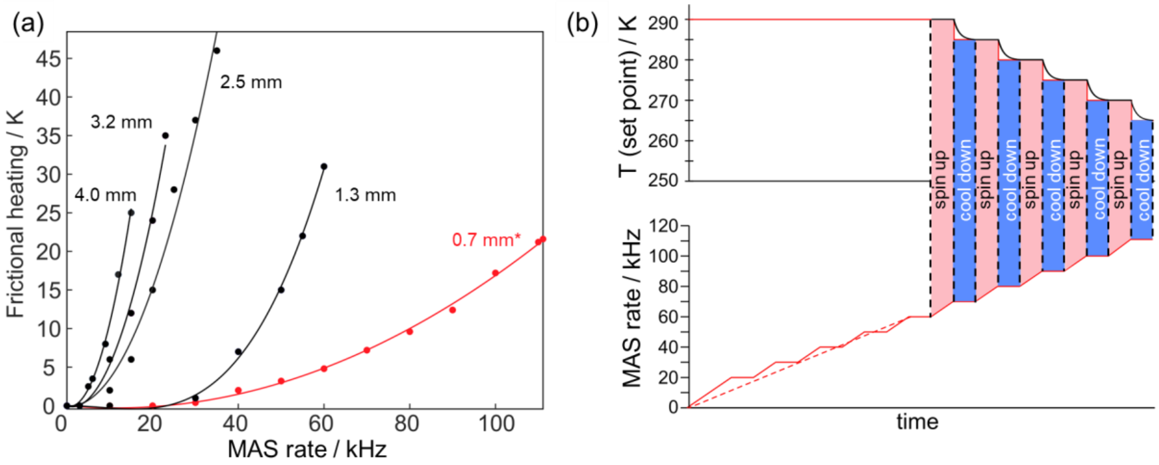

Figure S3. (a) Frictional heating expected for rotors with different outer diameters due to MAS at various spinning frequencies. Solid lines represent parabolic fits. Experimental datasets were determined by monitoring the ^{207}Pb chemical shift of lead chloride.[3] Data points for the 0.7 mm probe (marked with an asterisk) are inferred from the ^{79}Br chemical shift of KBr as described elsewhere.[4] In this experiment, the variable-temperature gas flow was set to 1200 L/h and regulated at 293 K. (b) Spin-up and cool-down method to cope with frictional heating in order to keep the sample temperature constant at about room temperature in Bruker 0.7 mm rotors. Based on Figures 19 and 20 in reference[5].

Lastly, we would like to note that for ultra-fast spinning, the gas supply for 0.7 mm rotors generally follows standard specifications used for other MAS probes. The recommended gas pressure is above 7 bar, ideally from boiled-off nitrogen. If using compressed air, it must be oil-free. The dew point should be between -60 and -80°C, but for optimal performance with 0.7 mm rotors, a dew point closer to -90°C is preferred. These specifications ensure that the gas supply setups used for larger MAS rotors are also suitable for fast spinning with 0.7 mm rotors.

Furthermore, 0.7 mm rotors can generally be safely spun up directly in the probe. Therefore, the use of a test spinning station is not mandatory for the application of ultra-fast MAS, and can be used based on personal preference.

S3. Experimental NMR Parameters

Table S1. ^{1}H Adiabatic Double Echo (ADE) NMR Experiments on $Na_3[Tb(DPA)_3]$ in **Figure 1**.

^{1}H Frequency (MHz)	500.13		
Recycle Delay (ms)	0.01		
Number of Scans	8192		
Acquisition Time (µs)	562.5		
SHAP Shape Parameter ζ	10		
SHAP Frequency Parameter tan(κ)	20		
SHAP Sweep width (MHz)	10, 5, 10		
Probe Representation	2.5 mm	1.3 mm	0.7 mm
MAS rate (kHz)	30	60	111
Rf-Amplitude (kHz)#	100	200	435
SHAP-Pulse Length (rotor periods)	1	2	2

#used for conventional excitation and adiabatic inversion pulses

Table S2. ^{1}H ADE NMR Experiments on Fe(py-NMe-PiPr$_2$)Cl$_2$ in **Figure 2a**.

^{1}H Frequency (MHz)	500.13			
Recycle Delay (ms)	6.5			
Number of Scans	65536			
Acquisition Time (ms)	300			
Rf-Amplitude (kHz)#	300			
SHAP-Pulse Length (µs)	18			
SHAP Shape Parameter ζ	10			
SHAP Frequency Parameter tan(κ)	20			
SHAP Sweep width (MHz)	10			
Probe Representation	3.2 mm	2.5 mm	1.3 mm	0.7 mm
MAS rate (kHz)	20	35	60	111
Echo Delay (µs)	16	5.29	7.67	9.02
Excitation-Acquisition Delay (µs)$	100	57.14	66.67	72.08

#used for conventional excitation and adiabatic inversion pulses
$delay between excitation and acquisition due to the adiabatic double echo sequence, affected by restrictions of the pre-scan delay

Table S3. ^{1}H Adiabatic Magic-Angle Turning NMR Experiments on Fe(py-NMe-PiPr$_2$)Cl2 in **Figure 2b**.

^{1}H Frequency (MHz)	500.13
Recycle Delay (ms)	5
Number of Scans	24576
Acquisition Time (µs)	300
Rf-Amplitude (kHz)$^{\#}$	400
MAS rate (kHz)	111
aMAT delay (µs)	19/6
Number of complex increments$^{\$}$	100
Spectral width in the direct (indirect) dimension (MHz)	1.875 (0.584)
SHAP-Pulse Length (µs)	18
SHAP Shape Parameter ζ	10
SHAP Frequency Parameter tan(κ)	20
SHAP Sweep width (MHz)	10

$^{\#}$used for conventional excitation and adiabatic inversion pulses
$^{\$}$echo/antiecho-acquisition scheme[6] was used

Table S4. ^{31}P ADE NMR Experiments on $LiFe_{0.25}Mn_{0.75}PO_4$ in **Figures 3** and **4.**

^{31}P Frequency (MHz)	121.495 / 202.457	
Recycle Delay (ms)	10	
Number of Scans	1048576	
Acquisition Time (µs)	100	
Rf-Amplitude (kHz)$^{\#}$	355 / 420	
SHAP Shape Parameter ζ	10	
SHAP Frequency Parameter tan(κ)	20	
Probe Representation	1.3 mm	0.7 mm
MAS rate (kHz)	60	111
Echo Delay (µs)	16.67	9.02
Excitation-Acquisition Delay (µs)$^{\$}$	100	72.08
SHAP-Pulse Length (µs)	16.67	18
SHAP Sweep width (MHz)	5	10

$^{\#}$used for conventional excitation and adiabatic inversion pulses
$^{\$}$delay between excitation and acquisition due to the adiabatic double echo sequence, affected by restrictions of the pre-scan delay

Table S5. ^{31}P ADE NMR Experiments on $LiMg_{0.5}Mn_{0.5}PO_4$ in **Figures 3** and **4.**

^{31}P Frequency (MHz)	121.495 / 202.457	
Recycle Delay (ms)	10	
Number of Scans	2097152	
Acquisition Time (µs)	100	
Rf-Amplitude (kHz)#	355 / 420	
SHAP Shape Parameter ζ	10	
SHAP Frequency Parameter $\tan(\kappa)$	20	
Probe Representation	1.3 mm	0.7 mm
MAS rate (kHz)	60	111
Echo Delay (µs)	16.67	9.02
Excitation-Acquisition Delay (µs)$	100	72.08
SHAP-Pulse Length (µs)	16.67	18
SHAP Sweep width (MHz)	5	10

#used for conventional excitation and adiabatic inversion pulses
$delay between excitation and acquisition due to the adiabatic double echo sequence, affected by restrictions of the pre-scan delay

Table S6. ^{31}P Adiabatic Magic-Angle Turning NMR Experiments on $LiFe_{0.25}Mn_{0.75}PO_4$ in **Figure *S9*.**

^{1}H Frequency (MHz)	121.495
Recycle Delay (ms)	10
Number of Scans	22528
Acquisition Time (µs)	300
Rf-Amplitude (kHz)#	355
MAS rate (kHz)	111
aMAT delay (µs)	28/6
Number of complex increments$	310
Spectral width in the direct (indirect) dimension (MHz)	1.875 (0.615)
SHAP-Pulse Length (µs)	9
SHAP Shape Parameter ζ	10
SHAP Frequency Parameter $\tan(\kappa)$	20
SHAP Sweep width (MHz)	10

#used for conventional excitation and adiabatic inversion pulses
$echo/antiecho-acquisition scheme[6] was used

S4. Sensitivity of 100+ kHz MAS for Paramagnetic Solids

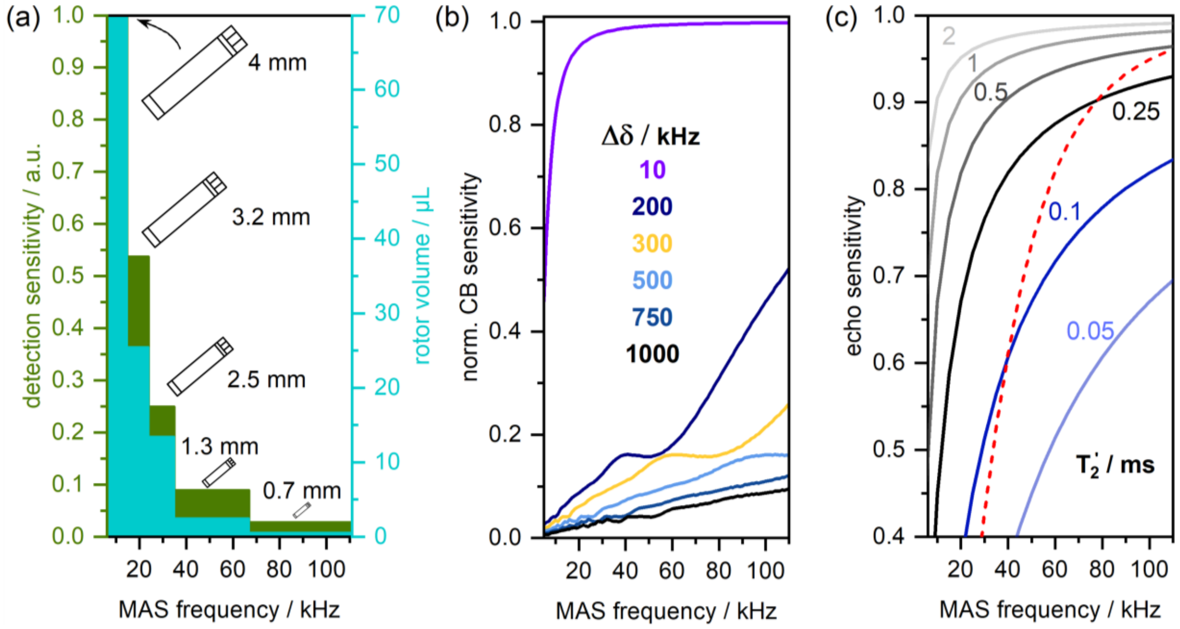

Figure S4. The different factors used to derive the normalized echo intensity for powdered paramagnetic solids at different spinning speeds, shown in **Figure 1b** of the main text. (a) Comparison of the rotor volume and detection sensitivity for commercially available Bruker rotors with diameters ranging from 4 to 0.7 mm. The detection sensitivity scales approximately with $\sim d^2$, where d is the inner rotor diameter.[5] The respectively available MAS rates are shown on the x-axis. The detection sensitivity for a 4 mm probe has been set to one. (b) The centerband intensity of MAS NMR signals with different shift anisotropies $\Delta\delta$. The asymmetry parameter is set to $\eta = 1$, so that the width of the spinning sideband manifold is $2\Delta\delta$. A CB intensity of one represents the absence of shift anisotropy. In (b), the inhomogeneous linewidth has been assumed to be identical, and independent of the MAS frequency for all signals. (c) The remaining signal intensity after two rotos periods (echo intensity) against the rotation frequency for different homogenous transverse relaxation times T_2'. The dashed red line shows the echo intensity for a MAS-frequency-dependent T_2', representing homonuclear-coupled networks, as e.g., formed by protons. Here, T_2' varies according to a quadratic function from 50 to 500 μs when increasing the rotation from 5 to 111 kHz.[5] The dashed red line has been used to computed the normalized echo intensities shown in **Figure 1b** in the main text. An intensity of one represents no intensity losses due to T_2' relaxation. For (b) and (c), ideal rf-pulses have been assumed.

S5. ^{1}H MAS NMR Spectra of $Na_3[Tb(DPA)_3]$ at Various RF Fields

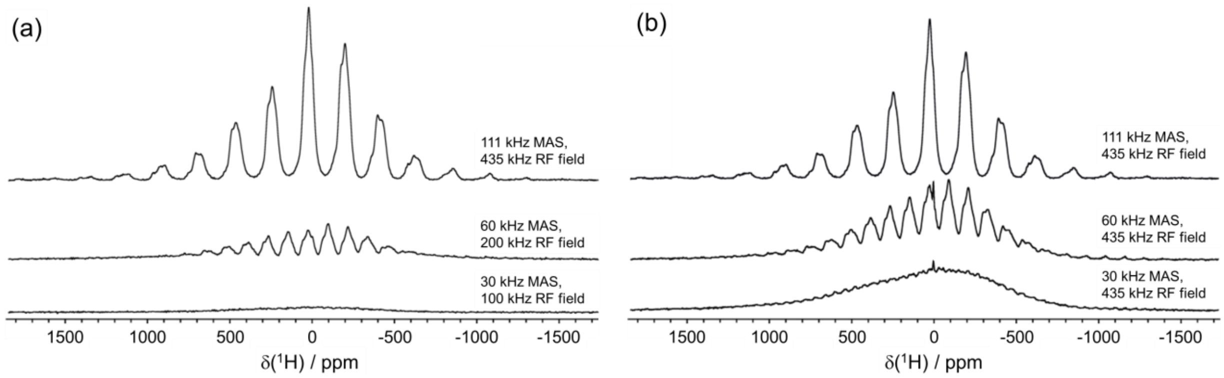

Figure S5. ^{1}H MAS NMR spectra of $Na_3[Tb(DPA)_3]$ obtained at 11.75 T, at spinning frequencies from 30 to 111 kHz. The spectra were acquired using a double spin-echo employing a conventional $\pi/2$ excitation pulse and a pair of short high-power adiabatic pulses, with (a) RF-field strengths representative of the maximum applicable for a 2.5 mm, 1.3 mm, and 0.7 mm probe, and (b) identical RF-field strengths of 435 kHz. (a) is identical to **Figure 1**a in the main text, and reproduced here for convenience. The sharp signal component at about 0 ppm visible in (b) at 30 and 60 kHz MAS is due to water (conducted later than the experiments in Figure 1a, these measurements reflect water absorption due to the sample's hygroscopic nature).

S6. 2D aMAT NMR Spectra of $Fe(py\text{-}NMe\text{-}PiPr_2)Cl_2$ at 60 and 111 kHz MAS

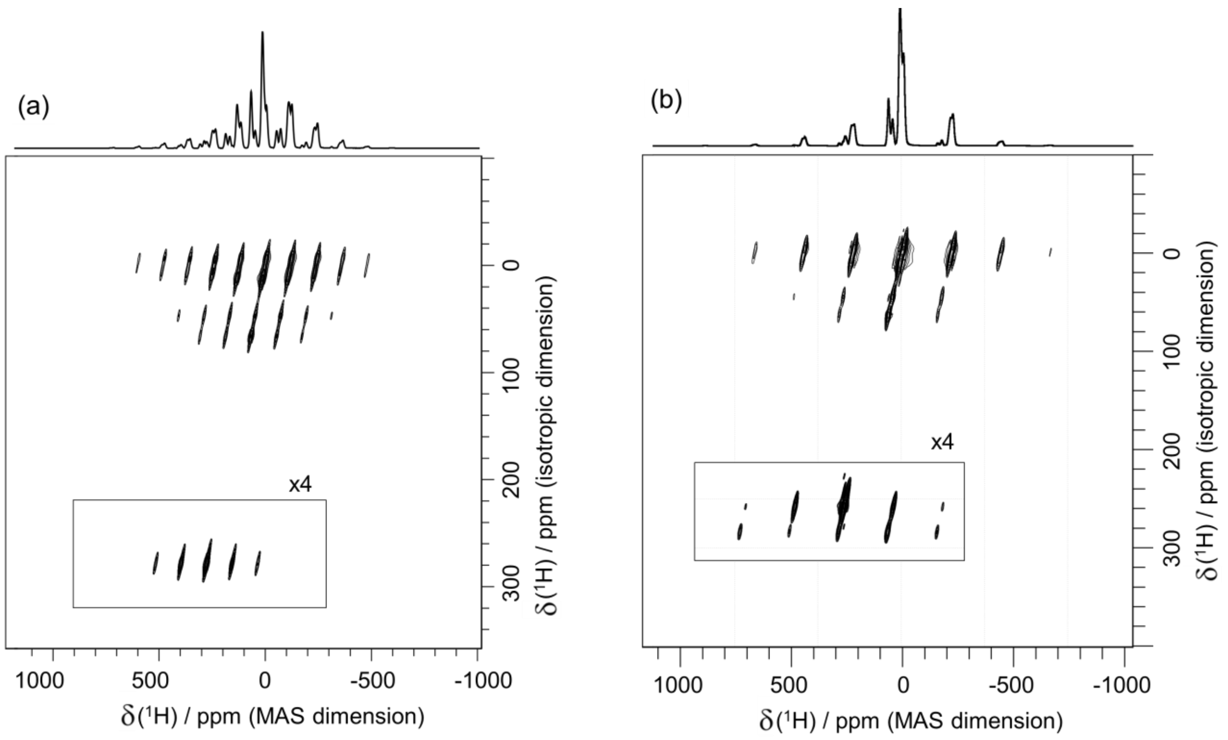

Figure S6. 2D aMAT NMR spectra of $Fe(py\text{-}NMe\text{-}PiPr_2)Cl_2$ obtained at 11.75T and (a) 60 kHz MAS, and (b) 111 kHz MAS. Signal traces shown in **Figure 2b** in main text are extracted from (b).

S7. Spectral Filter for 2D aMAT NMR Spectra at 100+ kHz MAS

The ^1H 2D aMAT NMR spectrum of Fe(py-NMe-PiPr$_2$)Cl_2 obtained at 11.75 T and 111 kHz MAS is shown in **Figure S8**a. As discussed in the main text, some of the signals are missing, e.g., the signal at -37 ppm due to the para-proton, which can however be observed in the ^1H MAS spectrum at 111 kHz (**Figure 2a** in main text). The reason why some of the weaker signals cannot be observed in 2D aMAT spectrum are superimposed artifacts stemming from the much more intense signals of the methyl-protons, as demonstrated by the horizontal trace extracted at -37 ppm in **Figure S8**b. However, these artifacts can be filtered out considering the specific structure of aMAT data. The complex, 2D time-domain signal can be written as[7]

$$S(t_1, t_2) = \sum_k s_k \exp[-i\omega_{iso}\, t_1] \exp[-i(\omega_{iso}\, t_2 + k\omega_{\mathrm{rot}} t_2)], \qquad (1)$$

where $\omega_{\mathrm{rot}}/2\pi = \nu_{\mathrm{rot}}$ is the rotation frequency, and s_k denotes the intensity of the kth sideband.[8] While the direct (t_2) dimension shows the typical MAS signal (isotropic and anisotropic chemical shift), the indirect (t_1) dimension is modulated by the isotropic chemical shift only. In the corresponding 2D aMAT frequency spectrum $S(\nu_1, \nu_2)$, in each individual horizontal trace $S(\nu_1', \nu_2)$, meaningful NMR signal will therefore only occur at $\nu_2 = \nu_1' + n\nu_{\mathrm{rot}}$, where n is an integer. This inherent feature can be exploited by an appropriate filter, which can in principle be applied in either the time or frequency domain, where we implemented the latter version here. Therefore, we computed a 1D pulse train $f(\nu_2; \nu_c)$ with periodicity ν_{rot}, and ν_2 covering the spectral width of the direct aMAT dimension. ν_c indicates the center of the pulse train. The width of each pulse can be chosen to maximize the reduction of artificial signals (and noise), but must be broader than the homogeneous linewidth $\frac{1}{\pi T_2'}$. A pulse train with a pulse width of $\frac{\nu_{\mathrm{rot}}}{3}$ is shown in **Figure S7**. In order to obtain a 2D aMAT filter, a series of pulse trains were computed, where ν_c was varied throughout the spectral window of the indirect aMAT dimension. Additionally, to further emphasize the NMR signal, each pulse was truncated using the Hanning window function.[9] The 2D representation is given in **Figure S8**c. The ridge of the center pulses at ν_c (shown in red) appears along the diagonal $\nu_1 = \nu_2$. The effect of multiplying the original aMAT spectrum with the 2D filter is demonstrated explicitly in **Figure S8**d for the horizontal trace extracted at -37 ppm. The corresponding 1D pulse train is likewise indicated.

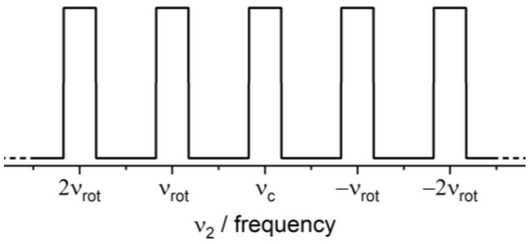

Figure S7. 1D pulse train $f(\nu_2; \nu_c)$ with periodicity ν_{rot}, centered at ν_c. Each pulse has a width of $\frac{\nu_{\mathrm{rot}}}{3}$.

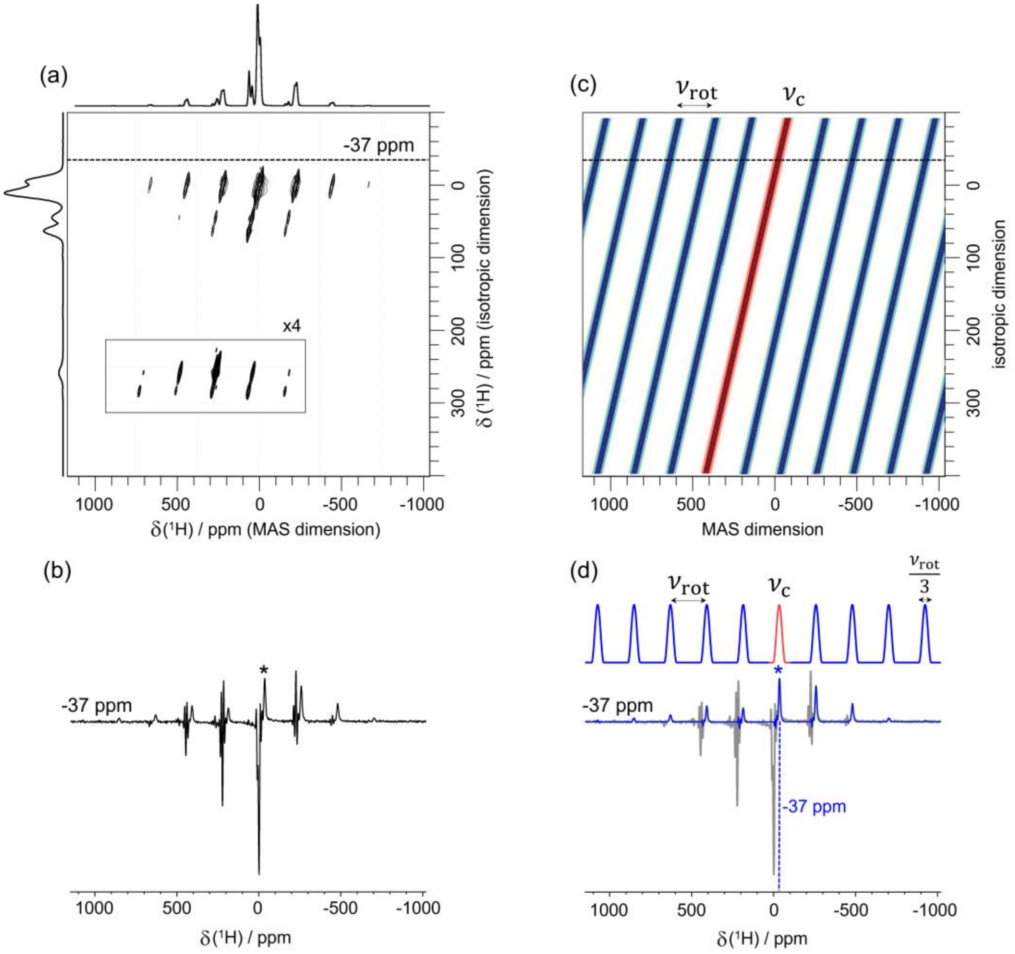

Figure S8. Application of the spectral filter designed for 2D aMAT NMR spectra at fast spinning frequencies. (a) ^{1}H 2D aMAT NMR spectrum of Fe(py-NMe-PiPr$_2$)Cl$_2$ obtained at 11.75 T and 111 kHz MAS. The isotropic dimension is the "infinite-MAS" spectrum shown in **Figure 2b** in main text. (b) Horizontal trace extracted at - 37 ppm. (c) The 2D filter function computed as discussed above. The ridge of the center pulses at ν_c is shown in red. (d) Horizontal trace extracted at - 37 ppm after application of the spectral filter. The corresponding 1D filter function is additionally shown in above the spectrum.

We note that while strong artefacts can mostly be removed by the application of the filter above, minor distortions of the extracted horizontal traces might remain. These can result in minor deviations between the observed signal and the corresponding numerical model, as e.g., the case for the ortho proton, (δ_{iso} = 109 ppm) in **Figure 2b** in the main text.

S8. Residual Sideband Intensity for $LiFe_{0.25}Mn_{0.75}PO_4$ at 111 kHz MAS

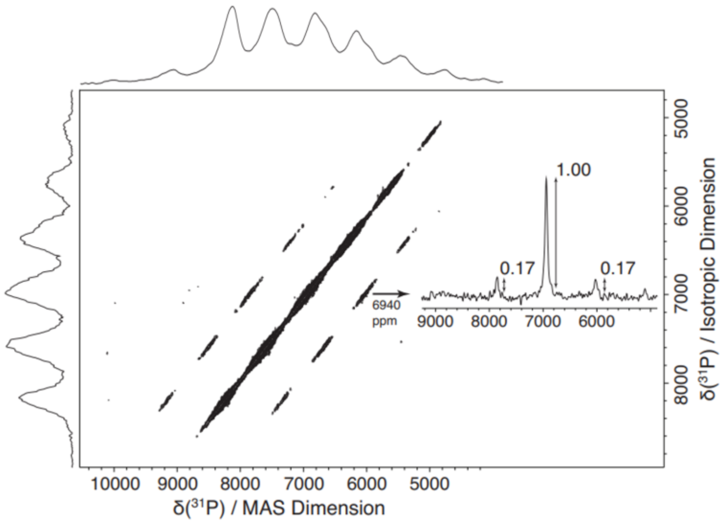

Figure S9. The 2D ^{31}P aMAT spectrum of $LiFe_{0.25}Mn_{0.75}PO_4$ acquired at an external magnetic field strength of 7.05 T and 111 kHz MAS. The spectrum indicates that the 1D MAS spectrum is largely composed of resonances corresponding to the isotropic shifts of the 32 sites present in the material, with small but non-negligible intensity in the first-order sidebands of each resonance. The cross section from 6940 ppm was used to constrain the sideband intensity at 17% of the centerband intensity in the fitting routine. All experimental parameters are summarized in **Table S6**.

S9. Routine for Modelling the ^{31}P NMR Spectra of $LiMg_xMn_{1-x}PO_4$

As described in the main text, the ^{31}P NMR shift of the 32 local ^{31}P environments in $LiMg_xMn_{1-x}PO_4$ is dominated by the Fermi-contact shift δ^{FC}, which can be approximated as the sum of all possible spin-transfer pathways to ^{31}P, that is, pathways of the type M-P comprising a single bridging oxygen, i.e., M–O–P, which are labeled $\varepsilon_1 - \varepsilon_4$ in **Figure S10**. This results in a measured shift of

$$\delta^{FC} = \varepsilon_1 + \varepsilon_2 + \varepsilon_{2'} + \varepsilon_3 + \varepsilon_4. \quad (2)$$

The routine used to fit the spectrum in **Figure S11** is listed below with the following assumptions:

- That the intensities of each of the 32 sites in $LiMg_{0.2}Mn_{0.8}PO_4$ are constrained assuming a statistically random distribution of metal ions around PO_4, i.e., the integrated area of each peak follows the distribution $0.2^m(0.8)^n$ for a configuration with Mg^{2+} ions and Mn^{2+} ions.
- That the intensities are further weighted by the expected T_2' relaxation losses during the pulse sequence, assuming that the ^{31}P T_2'is dominated by local PRE effects from the 5 metal centers coordinating PO_4. The T_2' coherence lifetime of $LiMnPO_4$ was measured to be 131.9 µs. As such the relaxation rate, R_2', for each ^{31}P site is modeled to vary linearly from $0.2R_2$'($LiMnPO_4$) to R_2'($LiMnPO_4$) as the number of Mn^{2+} coordination PO_4 increases from 1 to 5. The T_2' of each site is then calculated from the reciprocal of R_2'. The intensity of the peak corresponding to the 5-Mg site is subsequently allowed to vary as its T_2' does not follow this trend.
- That the line width for each site increases linearly with increasing Mn^{2+} content, starting from a value of 166 ppm (FWHM), which is the linewidth measured for the peak at 843 in the ^{31}P spectrum of $LiMg_{0.5}Mn_{0.5}PO_4$, shown in **Figure 3g**, and increasing to a variable value for the all-Mn site.
- That the spin-transfer contributions from the 5 spin-transfer pathways ε_N accurately determine the ^{31}P shift, resulting effectively in 32 isotropic ^{31}P environments.
- That despite the use of lower fields and higher MAS rotation rates, spinning sidebands intensity must be accounted for, especially first-order sidebands. As such we include first order sidebands for each of the 32 sites with intensity fixed at 15% of the intensity of the centerband, assuming that the both sidebands of each line have equal intensities (cf. **Figure S9**). This value was chosen by iteratively fitting the spectrum with varying sideband intensity until the in-tensity of the +1-order sideband of the all-Mn site at ~8300 ppm was well-represented. This represents a disadvantage of

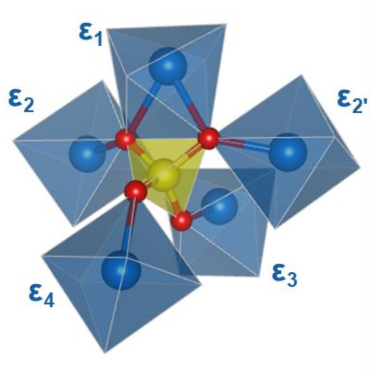

Figure S10. Local coordination environment of the P sites in $LiMPO_4$ (M is a divalent metal ion). The total ^{31}P shift is defined by the metal species occupying each of the five nearest neighbor metal sites and is given by the sum of five pathway contributions $\varepsilon_{1,2,2',3,4}$.

this method compared to the 2D sideband-separation methods employed by others, although in our opinions the ease of implementation of the experimental methods and high sensitivity of the 1D method is fair compensation.

- That the spin-transfer pathway of the Mn^{2+} atom contributing to the ε_1 pathway is constrained to 803 ppm, after scaling the value of the well-resolved peak at 843 ppm in **Figure 3g** to account for the different Weiss constant of this stoichiometry. This is a reasonable constraint, albeit not general, since the values calculated by Middlemiss et al.[10] shown in **Table S7** show that there is only one spin-transfer pathway contributing a one-metal shift in this range.
- That the 5-Mg peak, albeit weak, can be fit with a single Gaussian peak of variable width and position, which was constrained to be less 100 ppm.

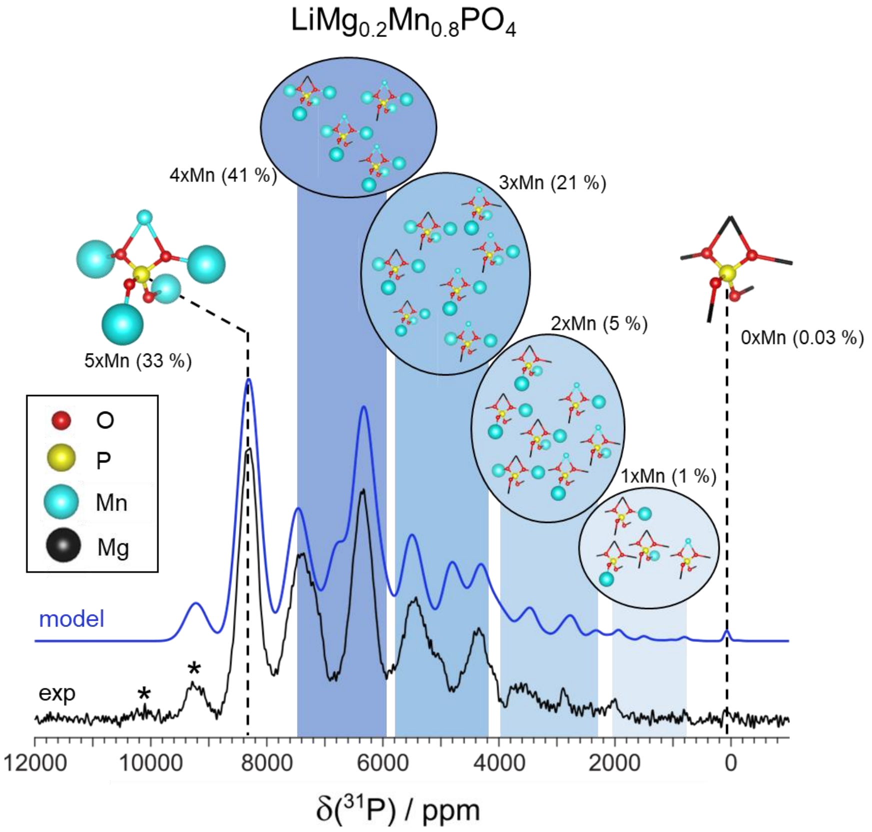

Figure S11. Complete characterization of the local P environments of mixed-composition olivine-type $LiMPO_4$ battery materials. ^{31}P MAS NMR spectra of $LiMg_{0.2}Mn_{0.8}PO_4$ obtained at 7.05 T and 111 kHz MAS and the assignment of the peaks as groups of resonances from the twenty-four distinct environments. In each case, the horizontal position of the P atom marks the assigned ^{31}P shift of the corresponding local environment. In the depiction of the local environments, the relative sizes of the spheres representing the metal ions reflect the corresponding absolute pathway contributions, i.e., the isotropic ^{31}P shift that is induced by replacing the diamagnetic Mg^{2+} with a Mn^{2+} at the respective position. The fractional populations of each group of local environments, assuming a random distribution of the two metal ions in the lattice, are given by the numbers in precent and match the signal integrals indicating that the composition is a solid solution.

The culmination of these constraints produces in a 7-parameter fit (the four ε_N pathways, not including ε_1, sideband intensity, linewidth of the 5-Mn peak, and shift of the 5-Mg peak), and the results of this fitting routine are given in **Figure S11**, with the extracted spin-transfer pathway contributions of Mn^{2+} compared against the DFT-calculated values in **Table S7**. In order to make a more meaningful comparison to the DFT-calculated values, the experimental values must be scaled according to the Weiss constant in the material. The Weiss constant in Ferro/Anti-Ferromagnetic describing the bulk materials can be calculated by summing over all Heisenberg exchange coupling constants $\mathcal{J}^{(AB)}$,[11]

$$\Theta = \frac{2S(S+1)}{3k_B} \sum_{\beta \neq \alpha} \mathcal{J}^{(AB)}. \quad (3)$$

In the case of $LiMg_{0.2}Mg_{0.8}PO_4$, this would produce unique local "Weiss constants" for each of the 32 possible structures. The determination of these constants without density functional theory (DFT) calculations is difficult, and therefore we have decided to model the Weiss constant of the bulk materials scaling linearly with Mn^{2+} content; for $LiMg_{0.2}Mg_{0.8}PO_4$, this results in an effective Weiss constant of Θ=46.4 K. The values in column 3 of **Table S7** are scaled to what would be expected for pure-phase $LiMnPO_4$ in column 4. Overall, the agreement between the results is impressive with all values falling near to those calculated, although there was an overestimate of the DFT-calculated value for ε_1 and a corresponding slight underestimate of the DFT-calculated value of ε_4.

Table S7*:* A summary of the DFT-calculated values for the spin-transfer pathways in $LiMnPO_4$ by Middlemiss et al.,[10] compared to the values extracted by fitting the spectrum of $LiMg_{0.2}Mg_{0.8}PO_4$ using the fitting routine given in the text. H20 and H35 correspond to DFT methods utilizing the B3LYP functional incorporating both 20% and 35% Hartree-Fock exchange, respectively. The experimental data were corrected for an expected difference in Weiss constants between pure-phase $LiMnPO_4$ and the mixed-phase $LiMg_{0.2}Mg_{0.8}PO_4$, given in the fourth column of the table. All values given in ppm. *Constrained to 803 ppm as discussed in the text.

Pathway	DFT (Middlemiss et al.[10])		Experimental	
	H20	H35	As fitted	Θ-Corrected
ε_1	671	544	803*	778
$\varepsilon_2 = \varepsilon_{2'}$	2245	1903	1941	1881
ε_3	1504	1219	1514	1468
ε_{14}	1996	1667	2107	2042

S10. References

[1] Y. Yang, J. Gurnham, B. Liu, R. Duchateau, S. Gambarotta, I. Korobkov, *Organometallics* **2014**, *33*, 5749–5757.

[2] J. Chen, M. J. Vacchio, S. Wang, N. Chernova, P. Y. Zavalij, M. S. Whittingham, *Solid State Ion.* **2008**, *178*, 1676–1693.

[3] G. M. Bernard, A. Goyal, M. Miskolzie, R. McKay, Q. Wu, R. E. Wasylishen, V. K. Michaelis, *J. Magn. Reson.* **2017**, *283*, 14–21.

[4] K. R. Thurber, R. Tycko, *J. Magn. Reson.* **2009**, *196*, 84–87.

[5] T. Le Marchand, T. Schubeis, M. Bonaccorsi, P. Paluch, D. Lalli, A. J. Pell, L. B. Andreas, K. Jaudzems, J. Stanek, G. Pintacuda, *Chem. Rev.* **2022**, *122*, 9943–10018.

[6] J. Keeler, D. Neuhaus, *J. Magn. Reson. 1969* **1985**, *63*, 454–472.

[7] I. Hung, Z. Gan, *J. Magn. Reson.* **2010**, *204*, 150–154.

[8] A. J. Pell, G. Kervern, L. Emsley, M. Deschamps, D. Massiot, P. J. Grandinetti, G. Pintacuda, *J. Chem. Phys.* **2011**, *134*, 024117.

[9] F. J. Harris, *Proc. IEEE* **1978**, *66*, 51–83.

[10] D. S. Middlemiss, A. J. Ilott, R. J. Clément, F. C. Strobridge, C. P. Grey, *Chem. Mater.* **2013**, *25*, 1723–1734.

[11] A. J. Pell, G. Pintacuda, C. P. Grey, *Prog. Nucl. Magn. Reson. Spectrosc.* **2019**, *111*, 1–271.
